# Spectral Power Density analysis of the resting-state as a marker of the central effects of opioid use in fibromyalgia

**DOI:** 10.1038/s41598-021-01982-0

**Published:** 2021-11-22

**Authors:** Maxciel Zortea, Gerardo Beltran, Rael Lopes Alves, Paul Vicuña, Iraci L. S. Torres, Felipe Fregni, Wolnei Caumo

**Affiliations:** 1 Laboratory of Pain and Neuromodulation at Hospital de Clínicas de Porto Alegre (HCPA), Ramiro Barcelos, 2350, Bairro Rio Branco, Porto Alegre, RS CEP 90035-003 Brazil; 2 Post-Graduate Program in Medical Sciences, School of Medicine, Universidade Federal Do Rio Grande Do Sul (UFRGS), Porto Alegre, Brazil; 3 Psychology Department, Universidad Catolica de Cuenca, UCACUE, Cuenca, Ecuador; 4 Institute of Neurosciences of the Universidad Catolica de Cuenca, UCACUE, Cuenca, Ecuador; 5 Pharmacology of Pain and Neuromodulation: Pre-Clinical Investigations Research Group, Universidade Federal Do Rio Grande Do Sul (UFRGS), Porto Alegre, Brazil; 6 Department of Pharmacology, Institute of Health Sciences (ICBS), Universidade Federal Do Rio Grande Do Sul (UFRGS), Porto Alegre, Brazil; 7 Post-Graduate Program in Biological Sciences: Physiology and Biological Sciences: Pharmacology and Therapy, Institute of Health Sciences (ICBS), Universidade Federal Do Rio Grande Do Sul (UFRGS), Porto Alegre, Brazil; 8 Berenson-Allen Center for Noninvasive Brain Stimulation (CNBS), Beth Israel Deaconess Medical Center and Harvard Medical School, Boston, USA; 9 Department of Epidemiology, Harvard T.H. Chan School of Public Health, Harvard University, Boston, USA; 10 Physical Medicine and Rehabilitation Department, Harvard Medical School, Boston, USA; 11 Laboratory of Neuromodulation, Spalding Rehabilitation Hospital, Harvard Medical School, Boston, USA; 12 Surgery Department, School of Medicine, Universidade Federal Do Rio Grande Do Sul (UFRGS), Porto Alegre, Brazil; 13 Pain Treatment and Palliative Medicine Service, Hospital de Clínicas de Porto Alegre, Porto Alegre, Brazil

**Keywords:** Neuroscience, Psychology, Biomarkers, Medical research

## Abstract

Spectral power density (SPD) indexed by electroencephalogram (EEG) recordings has recently gained attention in elucidating neural mechanisms of chronic pain syndromes and medication use. We compared SPD variations between 15 fibromyalgia (FM) women in use of opioid in the last three months (73.33% used tramadol) with 32 non-users. EEG data were obtained with Eyes Open (EO) and Eyes Closed (EC) resting state. SPD peak amplitudes between EO-EC were smaller in opioid users in central theta, central beta, and parietal beta, and at parietal delta. However, these variations were positive for opioid users. Multivariate analyses of variance (ANOVAs) revealed that EO-EC variations in parietal delta were negatively correlated with the disability due to pain, and central and parietal beta activity variations were positively correlated with worse sleep quality. These clinical variables explained from 12.5 to 17.2% of SPD variance. In addition, central beta showed 67% sensitivity / 72% specificity and parietal beta showed 73% sensitivity/62% specificity in discriminating opioid users from non-users. These findings suggest oscillations in EEG might be a sensitive surrogate marker to screen FM opioid users and a promising tool to understand the effects of opioid use and how these effects relate to functional and sleep-related symptoms.

## Introduction

Fibromyalgia is a syndrome characterized by generalized musculoskeletal pain, fatigue, muscle stiffness, sleep, and affective disturbances, as well as cognitive problems^1,2^. The pathophysiology of fibromyalgia is not fully understood. However, the increased brain responses found in functional neuroimaging experimental studies in patients with fibromyalgia lead to the hypothesis that may be due to dysfunctional cortical excitability^3^. The low efficacy of pharmacological treatment in primary chronic pain such as fibromyalgia is a challenge for clinicians. The impact of analgesic overuse in fibromyalgia, including opioids, is shown in a study involving six European countries and three Latin American countries, which found 70% of the patients were taking analgesics prescribed by physicians. Besides, proximally 40% of them were using over-the-counter painkillers. According to an earlier study, long-term opioid use in fibromyalgia is associated with worse pain outcomes than among opioid users without fibromyalgia diagnosis^4^. Long-term opioid use augments the dysregulation in the neurocircuitry involved in pain processing. Thus, an imbalance between the GABAergic and glutamatergic systems increased cortical excitability^5,6^. In chronic inflammatory and neuropathic pain conditions, GABAergic inhibitory control is reduced, leading to increased excitation and central sensitization^7^. In rats, morphine dose (3 mg/kg IP) produced a desynchronization. It decreased power in all frequency bands, except beta-2^8^. Besides, the prolonged exposure to opioids altered the structural plasticity in the long term even after stopping treatment, showing that drug abuse produces an ongoing reorganization of synaptic connection patterns^9^. Accordingly, opioids like morphine might induce a brain connectivity strength in the reward system. This dysregulation of circuits is associated with emotion and stress, enhanced beta and alpha power activity, and high impulsivity by Opioids Use Disorder (OUD)^10^. In contrast to other opioids, tramadol-related anti-nociceptive activity is not only mediated by mu-opioid receptors (MOR) but also results from serotonin and norepinephrine reuptake inhibition. Pre-clinical studies have pointed out that tramadol abuse deteriorates nervous tissues, including the cerebral cortex^11^. Hence, it has been shown that some symptoms of tramadol abuse are generated by neurotoxicity and cortical neurodegeneration^12,13^. Besides, earlier pre-clinical studies showed that tramadol-induced damage in the prefrontal cortex mainly through activation of neuro-inflammatory response^11^.

Considering the above mentioned, the assessment of cortical activity by electroencephalography (EEG) can provide information on the dynamics of brain activity by analyzing brain oscillations' spectral power^10,14^. Previous studies found increased either theta or beta frequency power band on the anterior cingulate cortex, prefrontal, and somatosensory in patients with neuropathic pain. In contrast, other studies found an increase in spectral power frequency in either migraine or complex regional pain syndrome^15,16^. In fibromyalgia, the lower alpha power band during the EEG with eyes closed compared to healthy subjects may be associated with diminished sensorimotor integration in brain processing^17^. Besides, they may be an indicator of a dysfunction in the cortical processing to attenuate the chronic pain sensation^18^. The choice for using the resting state with eyes open/eyes closed methodology is critical for stimulus-induced brain activation patterns. The activation of the ocular motor system and the deactivation of multiple sensory areas may go undetected with eyes open only. Recent research has explored this methodology in relation to attentional processes^19^. The main mechanisms is the “alpha desychronization” produced by exogenous manipulation of brain rhythms^20,21^. Due to the devastating effect of chronic use of opioids on the cortical excitability and function, it is relevant to explore different methodologies. To the best of our knowledge, no study was published so for aiming to address the use of opioids and eyes open *vs.* eyes closed variations (differences), although other methodologies to explore EEG spectral power density (SPD) have been implemented in opioid users. Findings point out to differences in alpha and beta bands for SPD^22^. Therefore, other wave bands besides alpha were also explored. Different levels of consciousness and cognitive activity can be associated with specific frequency bands. In this sense, broadly speaking, beta waves are associated with active and effortful cognitive activity, alpha with calmness and relaxed state, theta with deep relaxation and day dreaming, and delta with sleep, easily evident in the stages of sleep^23^.

Although exact neural mechanisms underlying opioid exposure have been involved in the increase in pain perception are scarce it is plausible that they might be related to cortical dysfunction evaluated by spectral power frequency. Thus, the main goal of this study was to compare EEG SPD of frontal, central, and parietal regions at rest between fibromyalgia subjects’ opioid users and non-users. We also tested if the variations between eyes open (EO) and eyes closed (EC) states could be a suitable marker to discriminate fibromyalgia subjects exposed and not-exposed to opioid, as well as to explore if these variations of the SPD were associated with disability due to pain, sleep quality, central sensitization, and other pain-related measures in fibromyalgia patients.

## Material and methods

### Design overview, settings

We conducted a cross-sectional study and used the Strengthening the Reporting of Observational Studies in epidemiology (STROBE) guidelines to report the methods and results. The study was approved by the Research Ethics Committee at the Hospital de Clínicas de Porto Alegre (HCPA) under the registration number 2017-0329) according to international ethical standards based on the Declaration of Helsinki. All participants were given written informed consent.

### Participants

We included women aged between 18 and 65 years, who can read and write, with a confirmed diagnosis of fibromyalgia according to the criteria of the American College of Rheumatology (2010–2016). They were recruited from the outpatient pain clinic of the HCPA Pain Service, the Basic Health Unit and via newspaper publicity. Volunteers were contacted by phone and invited for medical evaluation to confirm the diagnosis. Subjects were included if they were able to read, and they would report a score of at least six on the Numerical Pain Scale (NPS 0-10) on most of the time in the last six months. They were excluded if they had a history of alcohol or drug abuse in the last six months; neurological disease; history of head trauma or neurosurgery. They were also excluded if presented with decompensated systemic diseases, chronic inflammatory diseases (lupus, rheumatoid arthritis, Sjogren's syndrome, Reiter's syndrome), uncompensated hypothyroidism, under treatment for cancer.

### Instruments and assessment

The tools used to assess psychological and clinical measures were validated in the Brazilian population and three trained psychologists carried out the assessment, as well as the EEG recording and the psychophysical pain assessment.

#### Dependent and independent variable of main interest

**Figure 1:**
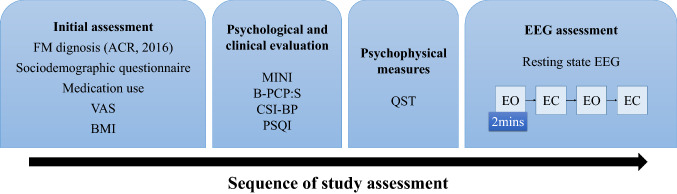
Flowchart of the study assessments. FM = Fibromyalgia; ACR = American College of Rheumatology; VAS = visual-analogue scale (10 cm) for pain levels; BMI = body mass index; MINI = Mini International Neuropsychiatric Interview; B-PCP:S = Brazilian Profile of Chronic Pain: Screen; CSI-BP = Central sensitization inventory—Brazilian Portuguese version; PSQI = Pittsburgh Sleep Quality Index; QST = Quantitative Sensory Testing; EO = eyes open; EC = eyes closed. Measures were not sequentially applied over time.

The dependent variable (outcome) was the spectral power density (SPD) by the peak amplitudes (EO-EC variations) evaluated by the delta, beta, alpha, theta EEG waves on the frontal, central, and parietal regions at resting state. The main interest factor was the exposure to opioids and non-exposure at the last three months when the subjects were classified as users and non-users. Other variables of interest were: age, years of study, pain catastrophizing thoughts, and disability due to pain, central sensitization symptoms, heat pain threshold, heat pain tolerance, sleep quality, psychiatric diagnosis and medication use. The timeline of assessments is presented in Fig. 1.

### EEG and resting state methodology

#### EEG recording

The EEG system consisted of the ENOBIO 20, Neuroelectrics (Barcelona, SP) and we used an EEG cap with circular gel electrodes with contact area of 1.75 cm^2^. EEG was recorded from 16 scalp sites (F7, F3, Fz, F4, F8, T7, C3, Cz, C4, T8, P7, P3, Pz, P4, P8, Oz,) and the left ear (EXT), referenced to the right ear (CMS/DRL). Artifact detection was implemented in two steps: first, detection of bad channels was made by visual inspection. Bad channels were excluded and interpolated using erplab’s spherical spline interpolation function. Secondly, automatic processes were implemented, as described below. Impedance was < 5 kΩ for all electrodes, with high dynamic resolution (24 bits, 0.05 uV) and sampling rate at 512 Hz.

### Resting state paradigm

There were four 2 min blocks of resting EEG collection: EO/EC/EO/EC to obviate EC/EO/ state differences due to resting time in the recording booth^21^. A brief calibration task was used for subsequent offline EO and EC correction of the EEG.

### EEG data preprocessing

EEG data were preprocessed using EEGLAB, an open-source toolbox running under the MATLAB environment (The MathWorks Inc., Natick, Massachusetts, United States). Continuous EEG data were band-pass filtered (1–45 Hz) using a Hamming finite impulse response filter (filter order: 3300; transition band width: 1 Hz; cutoff frequencies: 0.5–45.5 Hz). Then, Automatic artifact detection on epoched data: epochs with absolute threshold > or < 100uV were marked for exclusion. Channels with more than 40% epochs excluded by this criterion were interpolated. Frequency-domain data were obtained using the Welch method with a 500-point moving window.

### Estimation of resting-state EEG oscillation

Preprocessed EEG signals were transformed to the frequency domain using a Fast Fourier Transform (FFT, Welch algorithm, Hanning window, no phase shift, 0.5 Hz frequency resolution), yielding an EEG spectrum ranging from 1 to 45 Hz. For each participant, the spectra of each electrode were averaged across epochs. To identify the frequency intervals within which spectral power showed significantly different, we adopted point-by-point statistical analyses (e.g., for each frequency point) combined with a non-parametric permutation approach^24^. The spectral power density, measured at electrodes exhibiting the most prominent oscillation during wakeful rest^25,26^, was compared between for the opioid non-users and opioids users on each frequency point, thus yielding a map of values for each frequency point. To account for the multiple comparison problem in the point-by-point analysis, a cluster-level nonparametric permutation testing was performed^24^. Specifically, significant frequency points were categorized in clusters based on their frequency adjacency (cluster-level statistical analysis). Only the cluster with the largest number of significant frequency points was selected to control for false-positive observations. We performed 5000 random permutations, thus generating the cluster-level permutation statistics. The cluster-level statistics were defined by calculating the sum of the values of all frequency points within the cluster, and the two-tailed values were derived by locating the observed cluster-level statistics under the estimated permutation distribution. In order to confirm the identified frequency interval, a region of interest- (ROI-) based statistical analyses were further performed. Within this frequency interval, the average spectral power density within the frontal, central, parietal and temporal electrode ROIs (the electrodes for each ROI are summarized in Table 2) was computed for each participant. Electrode-level spectral power density within the alpha frequency band was compared between groups, using P-values based on paired Student t-tests. SD = standard deviation; ROI = region of interest.

#### Assessment of clinical, psychological, and psychophysical variables

(a) *Opioid exposure* Self-reported opioid use was considered the factor of interest assessed by a specific questionnaire that evaluated all medications used and their daily doses (e.g., antidepressants, anticonvulsant, hypnotic, analgesics non-opioid and opioid, etc.). Analgesic use was assessed by the self-reported average of analgesics used per week during the last three months. We defined opioid exposure using a categorical classification that combined information from pharmacy records of opioid medications dispensed and self-report information concerning recent opioid use. For data analysis, analgesic use was included as a dichotomous variable. We chose this strategy because subjects with chronic pain typically use rescue analgesics irregularly, and their frequency of use changes each day according to their pain level. Thereby, it would be difficult to quantify the precision of doses used to convert to morphine equivalent amounts and the exposure time. Thus, we used a simple based on the classification of opioid exposure or non-exposure in the last three months. The types of opioid medications used were classified according to classes [e.g., opium alkaloids (morphine, codeine), semisynthetic (oxycodone), or synthetic (methadone).

*(b) The American College of Rheumatology (ACR)*^1^ Criteria were used in physician-administered and patient self-administered questionnaires, increasing the correct diagnoses. The scale of fibromyalgia symptoms ranging from 0 to 3 and adding the widespread pain index (WPI) to the modified severity scale (SS scale), when administered to patients with and without fibromyalgia, using a score ≥ 13 as the cut-off point for positive and negative diagnosis, enabled a 93.0% correct diagnosis, with 96.6% sensitivity and 91.8% specificity.

*(c) Brazilian Profile of Chronic Pain: Screen (B-PCP: S)* The B-PCP:S consists of four questions related to pain severity, six questions related to pain's interference with functioning, and five questions related to emotional burden^27^. The pain intensity was assessed with a Numerical Pain Scale (NPS) score ranging from no pain (zero) to the worst possible pain (10). They were asked to answer the following question using the pain NPS: considering your pain, how intense was your worst pain during the most days at last 3 months.

*(d) The Pittsburgh Sleep Quality Index (PSQI)* The PSQI assesses sleep quality during the previous three months. It consists of 19 self-rated questions and five questions rated by the bed partner or roommate. These 19 items are grouped into seven component scores, each weighted equally on a 0–3 scale. The seven component scores are then summed to yield a global PSQI score, which has a range from 0 to 21; higher scores indicate worse sleep quality^28,29^.

*(e) The central sensitization inventory (CSI)* This tool identifies key symptoms related to central sensitization processes by quantifying the degree of these symptoms. It consists of two parts: Part A is a 25-item self-report questionnaire designed to assess symptoms related to health and Part B (not rated) is designed to determine if one or more specific disorders^30^.

*(f) Sociodemographic Questionnaire* Encompassed information related to age, years of study, body mass index (BMI), and questions related to clinical diagnoses, health problems (self-reported), and use of medication.

*(g) Mini-International Neuropsychiatric Interview (MINI*^31^*)* The MINI is a short (15–30 min) structured diagnostic interview aimed to screen for DSM-IV and ICD-10 diagnoses. In the present study, we reported information related to major depressive and maniac episodes, panic disorder, social phobia, obsessive–compulsive disorder, post-traumatic stress disorder, and generalized anxiety disorder.

*(h) Quantitative Sensory Testing (QST)* The QST with a computerized version of thermotest was used to determine the upper tolerance temperature and the heat pain threshold positioning the thermostat in the non-dominant ventral forearm. The temperature initiates at 32 °C and heated at a rate of 1.0 °C/s to a maximum of 52 °C. The participant reports the increase of the heat stimulus and presses the button when it was unable to tolerate it.

### Statistical analysis

Mean, standard deviation, frequency and percentage were used as descriptive analyses. Before inferential analyses, Shapiro-Wilks tests, histograms and box-plots for each group were used to verify data distribution and possible outliers. Sample characteristics between opioids groups (user and non-users) were tested using Independent sample *t-*tests or Fisher’s Exact Test.

We applied a full factorial two-way analyses of variance (ANOVAs), considering Group (opioid users and non-users) as a between-subjects factor and State (EO and EC) as a within-subjects factor. Dependent variables were delta, theta, alpha and beta SPD according to ROI (frontal, central and parietal). Partial eta squared (η^2^) effect sizes were reported (η^2^ = 0.01 indicates a small effect; η^2^ = 0.06 indicates a medium effect; η2 = 0.14 indicates a large effect)^32^ and pairwise comparisons were analyzed for the interaction term (Group *vs*. State). We applied this test to explore possible differences between EO and EC states. To test the main objective, we used EO-EC variations. Therefore, to analyze the EO-EC variations of peak amplitude of SPD in delta, theta, alpha and beta from frontal, central and parietal regions between groups (opioid users and non-users) were used the independent samples *t-*tests. Effect sizes were based on Cohen’s D coefficient (large effect is 0.8 or higher, a medium effect is 0.5, and a small effect 0.3^32^. To explore relations between peak amplitude (EO-EC variations) and pain-related measures, sociodemographic measures and sleep quality, Pearson’s *r* correlations were used. Subsequently, other potential factors that could explain variances in EO-EC variations based on correlations and sample differences were analyzed in an integrated multivariate analysis of covariance (MANCOVAs) model, where variations from EO-EC peak amplitudes of parietal delta, central theta, and central and parietal beta SPD were included as dependent variables, and Group (opioid users *vs*. non-users), age (due to the observed significant difference between groups), disability due to pain and sleep quality were factors. Multivariate and univariate statistics were considered and partial η^2^ were reported as effect sizes. For the univariate analyses, regression parameter estimates were reported to understand the contribution of Group and other factors. Finally, Receiver-Operator Curves (ROCs) with the area under the curve (AUC) as the discrimination index were plotted to verify if variations from EO-EC peak amplitudes would discriminate opioid users (identified as the positive cases) from opioid non-users (statistical test was based on differences from the null AUC = 0.5, or discrimination by chance, and based on non-parametric distribution). Sensitivity and specificity values were plotted and visually inspected for a plausible cut-off point with higher values. Significance level was considered when α < 0.05, for two-tailed tests. Software’s used were SPSS 21.0 (IBM Corp.) and JASP 0.12.2.0 (JASP Team, 2020).

## Results

### Sample characteristics

The sample was composed of the 61 patients that had EEG registered at rest; 13 (21.3%) had EEG registrations that did not pass the artifact detection test, and two were excluded by missing data. The final sample was formed of 47 patients. Sociodemographic and clinical characteristics are presented in Table 1. It was found that non-users were older and presented better quality of sleep than opioid users. Among opioids used, the most frequent were tramadol (73.33%) and codeine. (46.67%).

**Table 1:** Demographic and clinical characteristics of the study sample. Values are given as the mean (SD) or frequency (n = 47).

	Opioid users (n = 15)		Non-users (n = 32)		Between groups *p* values
	Mean	SD	Mean	SD	
**Sociodemographic and clinical measures**					
Age (years)	44.07	7.70	49.97	9.83	**0.047**
Years of formal study	11.80	3.32	12.61	4.85	0.562
Body mass index (BMI)	29.03	4.74	29.30	4.56	0.856
American College of Rheumatology (ACR) diagnostic tool (total score)	24.00	3.44	22.56	3.18	0.166
Worst pain during the last 3 months (Visual-analogue scale—[VAS]) for pain (score)	8.75	1.10	8.01	1.45	0.086
**Pain-related and sleep quality measures**					
Brazilian Profile of Chronic Pain: Screen (B-PCP:S) (total score)	75.33	10.89	69.80	14.17	0.188
Pittsburgh Sleep Quality Index (PSQI) (total score)	14.67	3.87	11.56	3.22	**0.006**
Brazilian Portuguese Central Sensitization Inventory (BPCSI) (total score) ^a^	68.20	13.62	61.68	13.57	0.134
Heat sensation threshold (HSTh) (°C)	33.27	1.16	33.42	1.04	0.675
Heat pain threshold (HPTh) (°C)	36.24	1.69	36.93	2.65	0.372
Heat pain tolerance (HPTo) (°C)	44.96	3.25	44.91	3.17	0.963
**Clinical comorbidity**					
HAS (Yes/No)	3/12	20.00	10/22	31.25	0.503
Cardiac disease (Yes/No)	0/15	0	2/30	0.67	1.000
Diabetes (Yes/No)	2/13	13.33	1/31	3.12	0.235
Hypothyroidism (Yes/No)	2/13	13.33	6/26	18.75	1.000
Asma (Yes/No)	4/11	26.67	5/27	15.62	0.438
Epilepsy (Yes/No)	0/15	0	0/32	0	0.319
Renal insufficiency (Yes/No)	0/15	0	1/31	3.12	1.000
**Psychiatric disorder**					
Major depressive episode (current) (Yes/No)	10/5	66.67	15/17	46.87	0.230
Manic episode (current) (Yes/No)	1/14	0.07	3/29	9.37	1.000
Panic Disorder (Yes/No)	4/11	26.67	6/26	18.75	0.648
Social Phobia (Yes/No)	3/12	20.00	5/27	15.62	0.697
Obsessive–Compulsive Disorder (Yes/No)	4/11	26.67	3/29	9.37	0.188
Post-traumatic Stress Disorder (Yes/No)	3/12	20.00	3/29	9.37	0.367
Generalized Anxiety Disorder (Yes/No)	1/14	0.07	10/22	31.25	1.000
**Medication use**					
Antidepressants (Yes/No)	11/4	73.33	20/12	62.50	0.527
Antipsychotics^a^ (Yes/No)	2/13	13.33	1/30	3.22	0.244
Anticonvulsant (Yes/No)	6/9	40.00	7/25	21.87	0.295
Benzodiazepines (Yes/No)	4/11	26.67	7/25	21.87	0.725
**Type of opioid**					
Methadone (Yes/No)	0/15	0	–	–	
Tramadol (Yes/No)	11/4	73.33	–	–	
Codeine (Yes/No)	7/8	46.67	–	–	
Oxycodone(Yes/No)	1/14	7.00	–	–	

Bold values indicates statistically significant differences (P < 0.05).

P-values for continuous variables (Sociodemographic, clinical measures and Pain-related and sleep quality measures) are based on Independent Student *t-*tests. Categorical variables (clinical comorbidity, medication use and type of opioid) are based on Fisher’s exact test. ^a^ = Measures from one non-user were not obtained, therefore n = 31.

### Univariate ANOVASs for peak amplitude of SPD according to Group of opioid analgesic exposure and EO/EC states

Table 2 presented the means, standard deviation and p-values for main effects and interactions for peak amplitude for each frequency band and ROI according to Group and State (EO/EC), based on ANOVA models. In relation to delta band, there was a main effect of State for the frontal ROI, indicating higher peak amplitude for EO compared to EC [F(1, 45) = 8.31; η2 = 0.16], and an interaction effect in the parietal ROI [F(1, 45) = 4.73; η2 = 0.09]. Pairwise comparisons indicated opioid users had a lower SPD with EC compared to EO (p = 0.048). Marginally significant, opioid users had a higher SPD for EO than non-users (p = 0.070). For the theta frequency band, SPD from were significantly higher in EC than EO state for frontal [F(1, 45) = 4.21; η2 = 0.09], central [F(1, 45) = 14.92; η2 = 0.25] and parietal [F(1, 45) = 14.00; η2 = 0.24]. Nonetheless, theta SPD from central site had a marginally non-significant interaction effect [F(1, 45) = 4.03; η2 = 0.08], pointing to an actual higher SPD in EC compared to EO only in the FM patients not in use of opioids (p < 0.001). This tendency was similar for parietal theta SPD (higher SPD in EC compared to EO only for the non-users group; p < 0.001), although the interaction term was non-marginally significant as well [F(1, 45) = 3.40; η2 = 0.07]. For the alpha frequency band, all sites had a higher peak amplitude for SPD regardless of Group: frontal [F(1, 45) = 45.15; η2 = 0.50]; central: [F(1, 45) = 54.35; η2 = 0.55]; and parietal: [F(1, 45) = 68.18; η2 = 0.60]. Finally, beta band presented a higher peak amplitude for SPD in frontal [F(1, 45) = 6.60; η2 = 0.13]; central [F(1, 45) = 11.70; η2 = 0.21] and parietal [F(1, 45) = 19.56; η2 = 0.30), and significant interactions between Group and State for central region (p = 0.015) and marginally non-significant for parietal region (p = 0.052). Pairwise scrutiny revealed only FM patients not in current use of opioids had higher peaks of beta in EC compared to EO state, both for central and parietal sites (p < 0.001). In addition, we observed a non-marginally significant effect in relation to Group (p = 0.061), meaning opioid users had a lower SPD peak amplitude at parietal ROI for EC state than non-users.

**Table 2:** Comparisons between Group (opioid users and non-users) and State (eyes open [EO] and eyes closed [EC]) for peak amplitude of SPD for each region of interest and frequency band (total n = 47).

Peak amplitude for ROIs		Opioid users (n = 15)				Non-users (n = 32)				Main effect of State	Main effect of Group	Interactions Group*State
		Eyes open		Eyes closed		Eyes open		Eyes closed				
		Mean	SD	Mean	SD	Mean	SD	Mean	SD	p	p	p
Delta	Frontal	11.9	3.8	10.4	3.1	10.4	2.8	9.1	2.3	**0.006**	0.063	0.865
	Central	9.2	2.2	8.5	2.4	8.2	1.6	8.0	2.4	0.179	0.208	0.354
	Parietal	11.4	4.7	10.2	4.5	9.1	3.6	9.5	3.7	0.246	0.209	**0.035**
Theta	Frontal	7.6	3.1	8.0	3.6	6.9	2.6	7.9	3.1	**0.046**	0.643	0.386
	Central	6.0	2.3	6.7	2.5	5.7	2.7	7.8	4.1	** < 0.001**	0.702	**0.051**
	Parietal	5.3	2.1	6.0	1.9	5.4	3.4	7.3	4.6	**0.001**	0.508	0.072
Alpha	Frontal	5.3	4.0	8.4	3.7	5.3	3.2	9.9	3.8	** < 0.001**	0.489	0.213
	Central	5.1	4.0	8.9	3.5	5.6	4.0	10.5	4.3	** < 0.001**	0.359	0.322
	Parietal	5.2	4.3	10.9	5.1	5.6	4.5	11.2	4.9	** < 0.001**	0.776	0.899
Beta	Frontal	1.6	3.6	1.8	3.2	1.5	3.1	2.6	3.0	**0.014**	0.743	0.065
	Central	0.9	3.1	1.2	2.7	0.9	2.8	2.7	3.0	**0.001**	0.386	**0.015**
	Parietal	0.9	3.0	1.6	2.4	1.5	2.8	3.4	3.3	** < 0.001**	0.162	**0.052**

Bold values indicates statistically significant effects (P < 0.05) and marginally non-significant effects.

P-values are based on univariate analysis of variance (ANOVAs). SD = standard deviation; ROI = region of interest.

**Figure 2:**
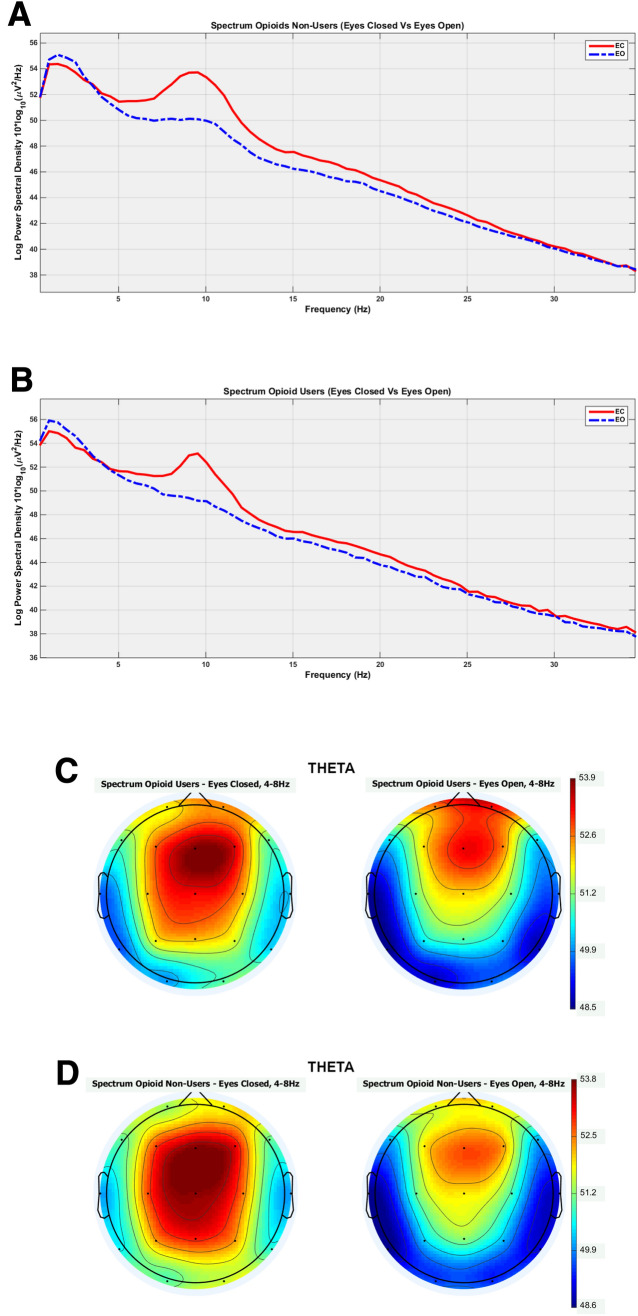
Spectral power density (SPD) to eyes close and eyes open states to opioid users (**A**) and opioid non-users (**B**). It shows significantly higher peak amplitudes for theta EC state compared to EO state for the non-opioid non-users. For opioid use groups, higher peak amplitudes for the EC state occur only in alpha band frequencies. Scalp topography maps for resting state for eyes closed (EC) and eyes open (EO) in the theta frequency bands to opioid users (**C**) and opioid non-users (**D**).

Figure 2 presents spectrograms and scalp maps according to ROIs and groups. In Fig. 2A one can view the spectrograms according to EO and EC states for opioid users and in Fig. 2B for opioid non-users. Figure 2C presents the scalp topographic maps for the SPD for the opioid users and Fig. 2D for opioid non-users. For parsimony reasons, only scalp maps for theta bands were presented. The scalp topography maps for the other frequency bands (delta, alpha and beta) are presented as supplementary material (Supplementary A).

### Between-group analysis of peak amplitude of SPD for ROIs according to Group of opioid analgesic exposure, considering variations between EO-EC states

Table 3 presents the comparisons between Groups (opioid users and non-users) for all ROIs considering EO-EC variations. The analyses revealed that patients in use of opioids had a smaller EO-EC variation for peak amplitudes in central theta, central beta and parietal beta, indicated by a mean closer to zero when compared to the non-users. For delta at parietal ROI the difference between groups was also significant, although in this case a positive EO-EC variation was found for opioid users. Trends indicating smaller EO-EC variations for opioid users were found as well for parietal theta and frontal beta band frequencies. Effect sizes were considered moderate for all differences, except for central beta that presented a large magnitude.

**Table 3:** Comparisons of peak amplitudes (variations between EO-EC states) for each ROI according to opioid users and non-users groups (total n = 47).

Peak amplitudes (EO-EC variations)		Opioid users (n = 15)		Non-users (n = 32)		*p*	Cohen's D
		Mean	SD	Mean	SD		
Delta	Frontal	1.22	2.54	1.28	2.94	0.946	− 0.02
	Central	0.73	2.12	0.14	1.98	0.354	0.29
	Parietal	0.85	1.25	− 0.36	2.24	**0.022**†	0.67
Theta	Frontal	− 0.39	1.17	− 0.97	2.44	0,274†	0.30
	Central	− 0.67	1.32	− 2.07	2.51	**0.016**†	0.70
	Parietal	− 0.67	1.61	− 1.86	2.18	0.068	0.59
Alpha	Frontal	− 2.90	2.77	− 4.60	3.76	0.126	0.49
	Central	− 3.77	3.68	− 4.96	3.83	0.322	0.31
	Parietal	− 5.74	4.87	− 5.57	4.13	0.899	− 0.04
Beta	Frontal	− 0.17	1.54	− 1.12	1.64	0.065	0.59
	Central	− 0.26	1.35	− 1.66	1.80	**0.010**	0.84
	Parietal	− 0.73	1.16	− 1.92	2.17	**0.018**†	0.69

Bold values indicates statistically significant differences (P < 0.05).

P-values are based on Independent Student *t*-tests, except for † which are based on Welch test due to heterogeneity of variance tested with Levene’s test. Effect sizes are based on Cohen’s D. ROI = region of interest; EO = eyes open; EC = eyes closed; SD = standard deviation.

### Relations between peak amplitude (variations of EO-EC states) age, pain-measures, sleep quality and psychophysical pain assessment

The correlation among the peak amplitudes for EO-EC variations with the pain measures, clinical and sociodemographic variables, despite the group of opioid users or non-user, are presented in Table 4. Data demonstrates EO-EC variations of peak amplitudes for beta at parietal region was negatively correlated to psychophysical pain measures (HPTh and HPTo). Peak amplitudes were negatively correlated to disability due to pain (B-PCP:S). Finally, positive correlations were observed between sleep quality (PSQI) with beta central and parietal amplitudes.

**Table 4:** Pearson's correlations between peak amplitudes (variations between EO-EC states), age, pain-related symptoms, sleep quality and psychophysical pain assessment (total n = 47).

Measures	Delta	Theta	Beta	
	Parietal	Central	Central	Parietal
Age	− 0.03	0.10	− 0.01	− 0.01
American College of Rheumatology (ACR) diagnostic tool (total score)	− 0.07	0.09	− 0.01	0.07
The worst pain during the most days at last 3 months (NPS) for pain (score)	0.02	− 0.05	0.21	0.10
Brazilian Profile of Chronic Pain: Screen (B-PCP:S) (total score)	− 0.30*	− 0.15	− 0.01	− 0.11
Pittsburgh Sleep Quality Index (PSQI) (total score)	0.05	0.26	0.41**	0.37**
Brazilian Portuguese Central Sensitization Inventory (BPCSI) (total score)	− 0.29	0.06	0.09	0.02

Measures of EEG are based on EO-EC variation of peak amplitudes. * p < 0.05; ** p < 0.01.

### Multivariate analysis of covariance (MANCOVAs) for SPD peak amplitudes at parietal delta, central theta, central beta and parietal beta EO-EC variations, according to group of opioid exposure, with disability due to pain, sleep quality and age as covariates

We used MANCOVAs to investigate the contribution of possible confounders to the effect of opioid use on SPD. In the multivariate model, we found main effect of Group [F(4, 39) = 3.56; p = 0.014; η2 = 0.27] and disability due to pain [F(4, 39) = 3.16; p = 0.024; η2 = 0.24]. Age and sleep quality did not have a significant effect considering the multivariate model. Nevertheless, for the univariate tests, where SPD peak amplitudes were considered separately, sleep quality was significantly associated with central beta [F(1, 42) = 4.92; p = 0.032; η2 = 0.10] and parietal beta [F(1, 42) = 5.37; p = 0.025; η2 = 0.11] models. Age, on the other hand, was not statistically significant yet. Table 5 presents statistical information for these models, based on parameters estimates. For the parietal delta (EO-EC variations) SPD peak amplitude, a group effect was observed (p = 0.022), as well as effect of disability due to pain (p = 0.017), indicating the effect of opioid use was still present regardless of the functional level. Factors in the model explained 13.4% of the parietal delta variance. Central theta was marginally non-significantly associated with Group (p = 0.057), as well as disability due to pain (p = 0.070). The model explained 12.5% of central theta variance. For the central beta, sleep quality was significantly associated with this EEG SPD (p = 0.032), and Group presented only a trend of association (p = 0.068). The model explained 17.2% of the dependent variable variance. Parietal beta was also associated with sleep quality (p = 0.025), and marginally non-significantly associated with disability due to pain (p = 0.78). For this EEG SPD, Group had no significant effect in the model, which explained 14.1% of parietal beta variance. Importantly, even though Group effect were marginally non-significant for some variables, B-values evidence it still contributed to a greater extent than disability due to pain or sleep quality.

**Table 5:** Parameter estimates of the Multivariate analysis of covariance (MANCOVA) model used to explain SPD peak amplitudes using Group, age, disability due to pain and sleep quality as factors (total n = 47).

Dependent variable EO-EC variations	Parameters	B	SE	t	P	95% Confidence Interval		Partial η^2^
						Lower bound	Upper bound	
Parietal delta R^2^ adj. = 0.134	Intercept	2.99	2.19	1.37	0.179	− 1.43	7.41	0.04
	Opioid users	**1.63**	0.69	2.37	**0.022**	0.24	3.01	**0.12**
	Non-users^a^							
	Age	0.01	0.03	0.41	0.684	− 0.05	0.08	< 0.01
	Disability due to pain^1^	− **0.06**	0.02	− 2.49	**0.017**	− 0.1	− 0.01	**0.13**
	Sleep quality^2^	− 0.01	0.09	− 0.13	0.900	− 0.19	0.16	< 0.01
Central theta R^2^ adj. = 0.125	Intercept	− 2.48	2.46	− 1.01	0.318	− 7.44	2.48	0.02
	Opioid users	**1.5**	0.77	1.95	**0.057**	− 0.05	3.06	**0.08**
	Non-users^a^							
	Age	0.04	0.03	1.23	0.224	− 0.03	0.11	0.03
	Disability due to pain^1^	− **0.05**	0.03	− 1.86	**0.070**	− 0.1	0	**0.08**
	Sleep quality^2^	0.13	0.1	1.35	0.185	− 0.07	0.33	0.04
Central beta R^2^ adj. = 0.172	Intercept	− 2.5	1.87	− 1.34	0.187	− 6.27	1.26	0.04
	Opioid users	**1.1**	0.58	1.88	**0.068**	− 0.08	2.27	**0.08**
	Non− users^a^							
	Age	0.01	0.03	0.45	0.652	− 0.04	0.07	< 0.01
	Disability due to pain^1^	− 0.02	0.02	− 1.25	0.219	− 0.06	0.01	0.04
	Sleep quality^2^	**0.16**	0.07	2.22	**0.032**	0.01	0.31	**0.1**
Parietal beta R^2^ adj. = 0.141	Intercept	− 1.87	2.1	− 0.89	0.379	− 6.12	2.38	0.02
	Opioid users	0.86	0.66	1.31	0.198	− 0.47	2.19	0.04
	Non-users^a^							
	Age	0.01	0.03	0.28	0.782	− 0.05	0.07	< 0.01
	Disability due to pain^1^	− **0.04**	0.02	− 1.81	**0.078**	− 0.08	0	**0.07**
	Sleep quality^2^	0.19	0.08	2.32	**0.025**	0.02	0.36	**0.11**

Bold values indicates statistically significant effects (P < 0.05) and marginally non-significant effects.

EO = eyes open; EC = eyes closed; SE = standard error. ^a^ = reference group for comparisons.

^1^= indexed by B-PCP:S = Brazilian Profile of Chronic Pain: Screen (total score).

^2^= indexed by PSQI = Pittsburgh Sleep Quality Index.

### ROC analyses to test the capability of the peak amplitudes (EO-EC variations) to discriminate opioid users from opioid non-users

**Figure 3:**
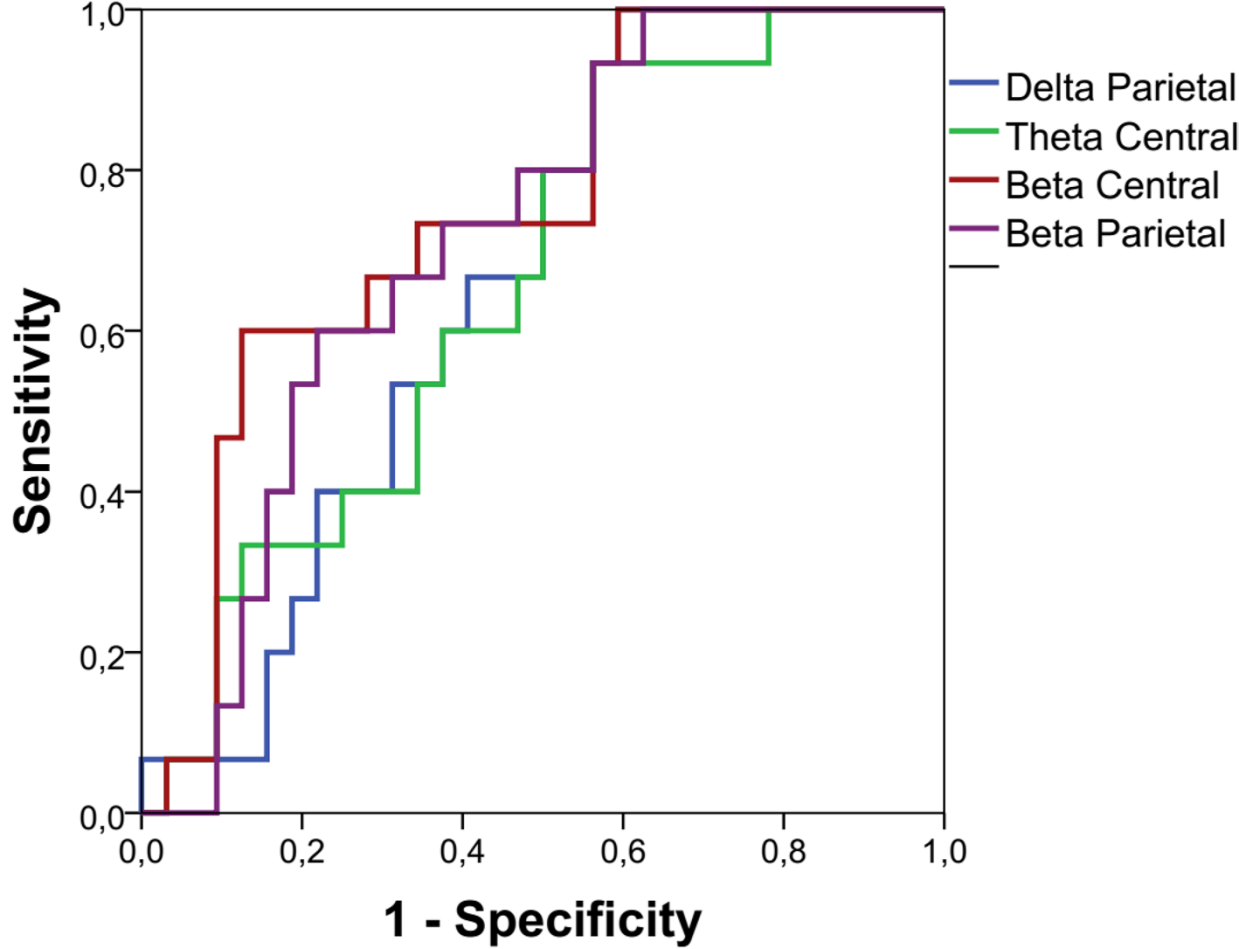
Receiver-operator curves (ROCs) for the parietal delta, central theta, central beta and parietal beta peak amplitudes ((EO-EC variation) of the Spectral power density (SPD).

To examine if the peak amplitudes (EO-EC variations) could discriminate opioid user from non-users, we tested ROCs for the ROIs that presented significant differences between groups. Figure 3 presents these curves. The beta central at the cut-off point of − 1.05 offers 67% sensitivity and 72% specificity to screen fibromyalgia opioids users than non-users. The AUC for beta central was equal to 0.750 (SE = 0.074; p = 0.006; 95% CI 0.605–0.895). The beta parietal at a cut-off point of − 1.28 offers 73% sensitivity and 62% specificity to screen fibromyalgia opioids users than non-users with an AUC was 0.717 (SE = 0.075; p = 0.018; 95% CI 0.570–0.863). The other two measures of SPD had a marginally non-significant trend to discriminate opioids users from non-users (delta parietal p = 0.075; theta central p = 0.083).

## Discussion

This study showed two main findings related to FM patients in use of opioid analgesics associated to the variations between EO and EC states on the oscillations of SPD in frontal, central, and parietal regions at resting: First, the variation for peak amplitudes EO-EC was smaller in opioid users than non-opioid users in the central theta, central beta, and parietal beta. At the parietal region, the delta EO-EC variation was positive for the opioid users. This indicates that in EO the peak amplitude was larger than EC. However, the EO-EC variations in parietal delta were also negatively related to the disability due to pain, and central and parietal beta were positively correlated with sleep quality. Second, these variations in EO-EC states for central beta and parietal beta are likely suitable markers to discriminate opioid users over non-users. In sum, these results give insights into generating testable hypotheses regarding the dynamics of resting-state brain activity that may disrupt opioid users.

These results give a remarkable contribution to this field of knowledge, indicating that opioid users lack expected neural modulation between EO and EC states. However, the exposure of opioid use has been assessed by self-reported, a factor that restricts us from making inferences about its relationship with dose or a specific drug. In contrast, the strength of these results is mainly because our primary outcome was evaluated by electrophysiological measures upon an experimental controlled condition, making them less prone to assessment biases. They are relevant to research and perhaps to the clinical setting, mainly to open an avenue to use the amplitude of EEG indexed by SPD as a marker to comprehend opioids' effect on cortical processing. Despite this contribution to the scientific field, we cannot isolate if these changes in the electrophysiological measures indicate a deteriorated cortical processing related to the severity of disease or consequence of opioids effects. In fact, recent findings suggest the severity of symptoms, including pain duration, anxiety and depression was negatively correlated with EEG wave power^14^). However, we need to realize that these are aspects intrinsic of the condition of illness (i.e., severity of symptoms and analgesic demand), which we cannot change.

Another exciting result is that we found these associations in a sample of FM, in which the most frequent opioid used was tramadol. Recently, tramadol has been attributed some potential benefits over other opioids on treating pain symptoms in fibromyalgia^33^. It is important to stress that there is no evidence about its effect based on clinical outcomes. Their theoretical benefits are associated with its pharmacodynamics properties, such as act a weak an um agonist mu, and efficacy is linked to action on serotonin reuptake inhibition and noradrenaline^33^. Based on these pharmacological properties, its reputation is growing as a drug with a more favorable side effect profile, including lower constipation rates, overdose, and addiction^34^. This way, these results based on neurophysiological measures are essential to understand the neural mechanisms responsible for its effect at the cortical level. Given there is growing evidence that excessive tramadol consumption causes significant changes in the prefrontal cortex's cognitive function^35,36^. Based on this data, it is plausible that tramadol use can increase the cortical dysfunction that leads to symptoms severity in fibromyalgia, which itself may induce the prescription of higher dose and more frequent analgesic. This way, the severity of pain can work as a trigger to use opioids, which on the other side contributes to change the cortical dysfunction, forming a vicious cycle that self feeds back. From this perspective, the current findings contribute to revealing a neural substrate to comprehend the harmful role of the opioid on the cortical neural networks.

Although the study design does not permit establishing a cause consequence relationship type, our results highlight that these electrophysiological findings are associated with higher disability due to pain and worse sleep quality. Although this finding should be taken cautiously due to multiple comparison limitation for the correlation analyses presented in Table 4, it may be important for understanding possible confounders and mediators of the relation between opioid analgesics use and EEG SPD. ANOVAs revealed that for alpha SPD (and frontal delta), peak amplitudes were smaller in EC compared to EO state independently of opioid use. Nevertheless, for theta and beta bands, we observed EC/EO differences only for the opioid non-users group, which once more point to less flexibility of the neural system to adapt to different states and stimuli. According to the literature, a decreased delta and theta are generally associated with stimulus processing and hence indicate decreased activation in the EO state. Nevertheless, differences between EO-EC distributed throughout the scalp are associated with general arousal levels^20^. Considering that we did not assess cognitive functions, we cannot affirm if this neurophysiological finding is associated with changes in neurocognitive mechanisms or if they relate to the arousal level of the whole nervous system. In addition, we found that specifically for beta waves at central and parietal regions, sleep quality was also a predictor of SPD along with use of opioids. From the functional point of view, this finding may be central to explain the association of opioid and the central electrophysiological signal, since opioid users have reported also a poorer quality of sleep. Other studies support the deleterious effect of opioid analgesics to sleep^37^.

According to the literature, lower peaks in the opioid users' EC state than EO might indicate mental fatigue characterized by exhaustion and loss of motivation provoked by excessive energy spent^38^. Thus, this smaller EO-EC peak amplitude variation in central theta, central beta, and parietal beta bands in our study might reflect a cortical dysfunction in opioids exposure indexed on SPD oscillations. This result finds support in an earlier study in elderly subjects, which found that a reduced attentional task performance accuracy was positively associated with decreased beta activation over the primary visual cortex, which might be correlated with the deterioration of attention^39^. Also, an earlier study found that a larger theta power in the frontal-central and parieto-occipital were associated with increased error rates in a long-duration cognitive task (4 h)^40^. While another study found that a preference color task increased the occipital theta activity by the possible effect of attention-related brain oscillatory activities^41^. Thus, this set of results indicates that theta activity might be correlated with an attempt to maintain attention and reaction time during cognitive tasks. Aligned with this perspective, our outcomes related to a decrease in EEG beta bands suggest that opioid user presents a diminished reaction to arousal and reduced attention. Accordingly, these results related to decrease in SPD in opioid user might be linked to a lower arousal level, which might be related to fatigue. This hypothesis is supported by an earlier study that found an association between similar results in the EEG measures with fatigue on acute opioid effect^40,42^. Decrease in SPD were also found in a study with long-term use of heroin^43^, in theta, delta and alpha frequencies, and an increase in beta, although the authors used only eyes-closed resting state. They interpret these findings as a neurophysiological indicator of brain atrophy and chronic brain damage in heroine addicted. This way, it remains unclear if the decreased theta amplitude variation in opioids users would be associated with mental fatigue in long-term opioid use or, when taking together the decreased SPD for delta, theta and beta, it could be a product of a more severe cortical dysfunction.

In interpreting our findings, it is essential to consider that 73.33% of opioid users were taking tramadol. Despite some used more than one opioid, it needs parsimony to generalize this result, mainly because we cannot affirm if this effect is a drug-specific or a class of drug effect. However, they are particularly important to explore potential consequences of long-term tramadol use since preclinical studies showed that either in acute or chronic use rats showed an impaired spatial memory capacity^44^. In humans, surveys found that subjects with tramadol abuse were about two times more likely to have cognitive impairment assessed by the Montreal Cognitive Assessment test (MOCA) than controls. They found that 96% of tramadol use presented a delayed recall^45^. Although full opioid agonists (e.g., morphine) impair some aspects of psychomotor and cognitive performance^46^, there is a low prevalence of use of other opioids in the present study without tramadol. Thus, this aspect restricts the generalizability of current findings for all opioid s classes.

The receiver operator characteristics (ROC) analysis was used to screen the opioids users than non-users, as observed in the EEG measures indexed by SPD in both beta central and parietal. This showed that the SPD beta central and beta parietal offers AUC higher than 0.71. This considerable change of cortical activation in opioid users suggests a more deteriorated function of the cortical processing. More precisely, from a conceptual perspective, our findings might explain the pathophysiological processes that underlie fibromyalgia since they integrate the severity of symptoms with dysfunctional changes in cortical areas involved in the disability due to pain and the worst sleep quality. These dysfunctions on cortical processing might help investigate the relationship between the deteriorated cortical processing in opioid users and if the therapeutic approaches can reverse this dysfunctional processing.

The main concerns related to the interpretation of these findings include several aspects: First, the cross-sectional design did not allow whether the electrophysiological changes represent a more severe disease, or a consequence of long-term chronic pain, or both, together with the use of the opioid, that it can inflate symptoms such as fatigue^47^. Second, the relationship between opioid use and oscillations in EEG indexed by SPD may be confounded by other factors, such as age or medication use. Besides, the intake of medication targeting the central nervous system might confound the electrophysiological indexed by the SPD. However, many potential confounders might not be completely controlled in patients with fibromyalgia, such as the medication – patients with fibromyalgia commonly use a mixture of antidepressants, analgesics, mood stabilizers, and antipsychotics. In research with fibromyalgia, polypharmacy is a challenge to control their confounding effect appropriately on the outcomes since the severity of the disorder prohibits medication as withheld for research purposes. Also, the variety of classes, the use of doses according to demand and the diversity of pharmacological targets difficult the interchange of dosage equivalent across opioids classes. Third, in the absence of other patient group comparisons, it is impossible to infer whether the differences seen relate specifically to fibromyalgia or more general aspects of chronic pain^48^. Fourth, although our findings reveal alterations in cortical areas, any relationship with pathophysiology is speculative. A clinical study is not feasible to direct thalamic activity recordings, which would confirm abnormal thalamocortical rhythms^49^. Fifth, to ensure participants remained awake and alert throughout recordings, and stringent control levels were applied during the assessment. It is still possible that other confounding factors such as drowsiness could unduly influence findings. Sixth, we excluded the analysis participants due to low signal quality in EEG measures' acquisition procedure according to filter procedure^50^. Another aspect related to the sample is that these findings are generalizable to women. We included only females because fibromyalgia is more prevalent in women, and pain processing is distinct in females and males^51^. Finally, we have worn that these results cannot be generalized to opioid abusers since took most parts of the opioid users were taken tramadol. If, on one side, the high prevalence of tramadol use is a limitation for the generalizability of other opioids, it gives support for the idea that the difference between users and non-users in neurophysiological measure can be related to tramadol. Thus, these results are manly relevant to open new avenues to investigate its effect in the cortical processing using less prone to assessment bias, and while give support for further studies to investigate the impact of therapeutic approaches in these neuropsychological measures and if their interplay with clinical outcomes.

In conclusion, this study provides additional evidence that the oscillations in EEG indexed by SPD might be a sensitive surrogate marker to screen fibromyalgia opioid users with more disability due to pain and more severe sleep disorders. Overall, they offer insight into comprehending the chronic opioid exposure by the SPD.

## Supplementary Information

Supplementary Information 1.

Supplementary Information 2.
