# Supplementary material for: Spectral Power Density analysis of the resting-state as a marker of the central effects of opioid use in fibromyalgia
Source: Sci Rep. 2021 Nov 22;11:22716. doi: 10.1038/s41598-021-01982-0 (PMC8608932; doi:10.1038/s41598-021-01982-0)
Supplement: Supplementary file 1 — Supplementary Information 1. [file 41598_2021_1982_MOESM1_ESM.docx]

**Supplementary A.** Scalp topography maps of resting state for eyes closed (EC) and eyes open (EO) states in the delta frequency bands to opioid users (A) and opioid non-users (B). For opioid non-user groups, significant differences between EC-EO states in higher peak amplitudes occur in frontal areas. For alpha frequencies, significant differences between EC-EO states in higher peak amplitudes occur in frontal, central, and parietal areas, in opioid users group (C), as well as, in opioid non-users group (D), that represents the suppression of alpha frequency bands during the EO state. For beta frequency bands, significant differences between EC-EO states in higher peak amplitudes occur in frontal, central, and parietal areas in the opioid users group (E), in contrast with the opioid non-users group (F) that present significant differences between EC-EO states in higher peak amplitudes in parietal areas solely.
