# Supplementary figures and images for: Spectral Power Density analysis of the resting-state as a marker of the central effects of opioid use in fibromyalgia
Source: Sci Rep. 2021 Nov 22;11:22716. doi: 10.1038/s41598-021-01982-0 (PMC8608932; doi:10.1038/s41598-021-01982-0)

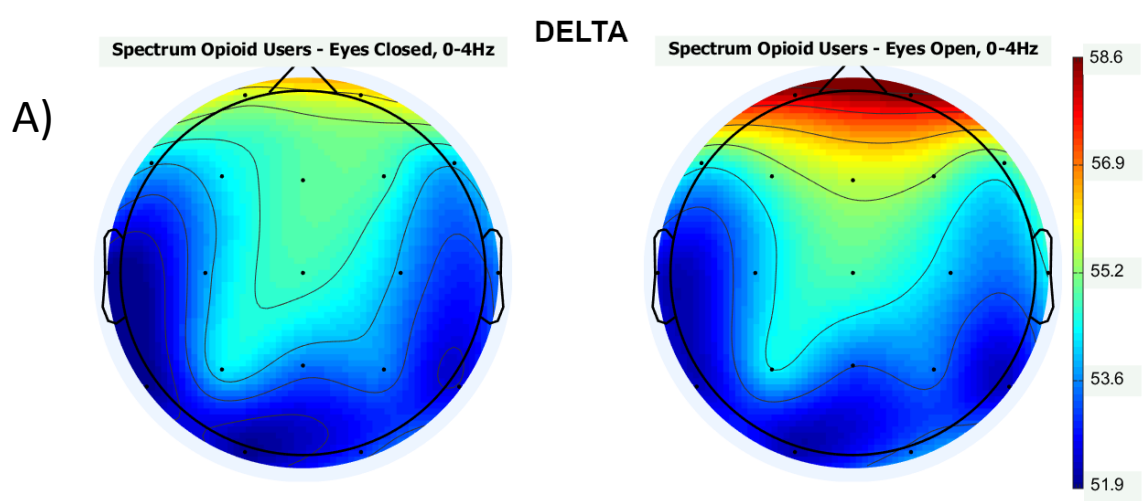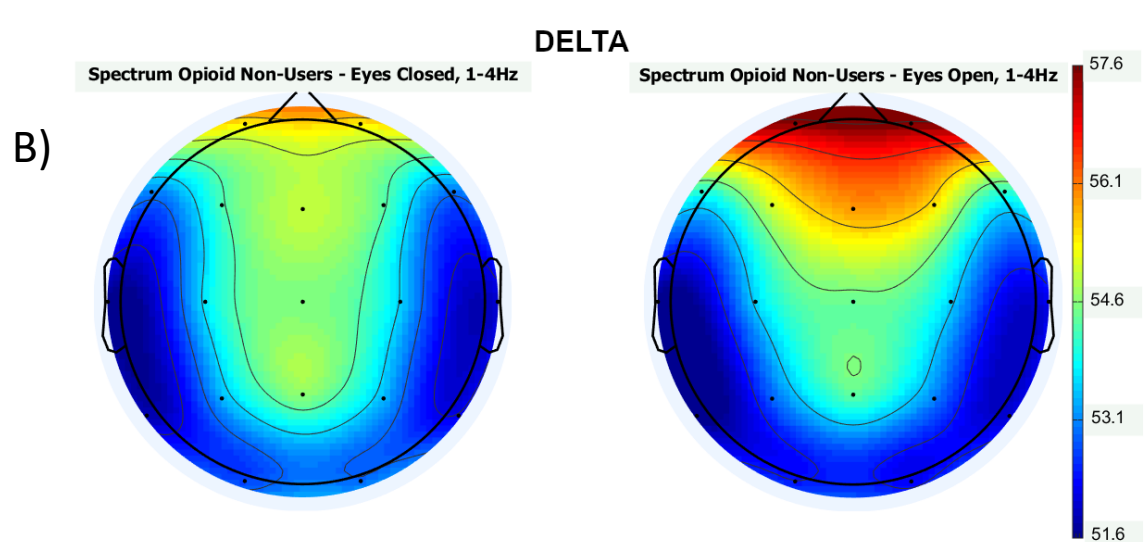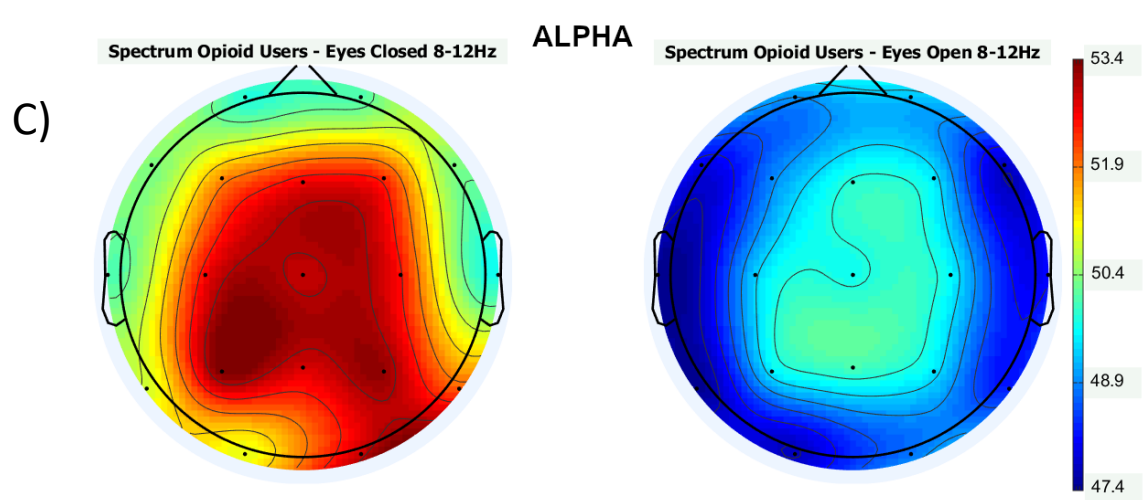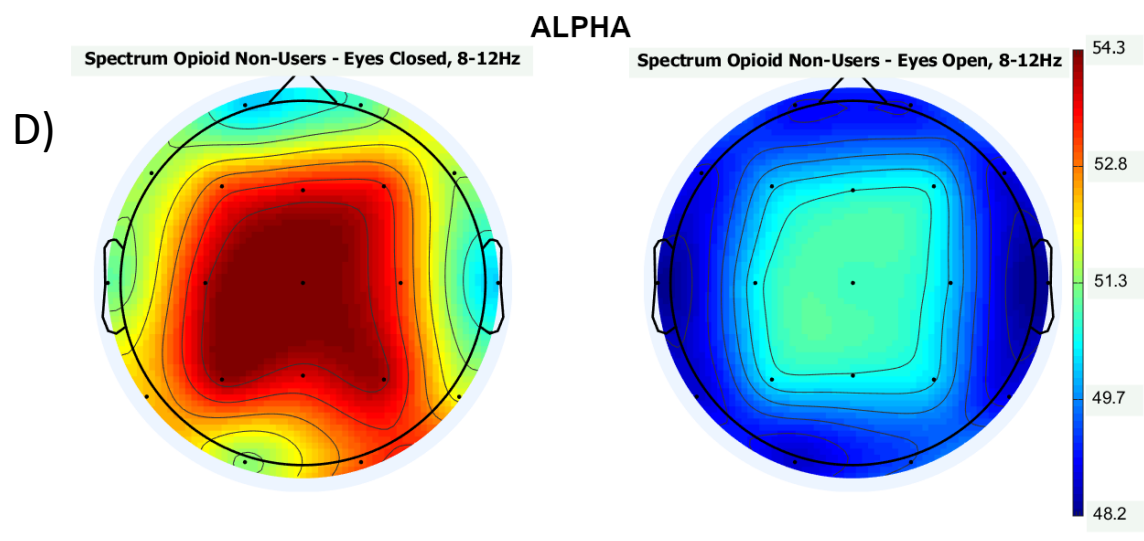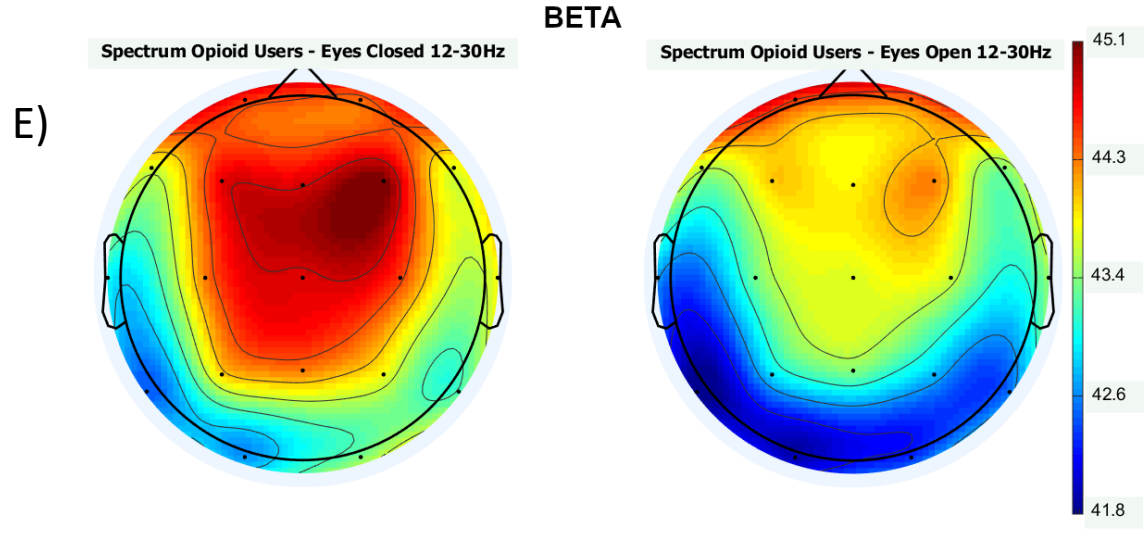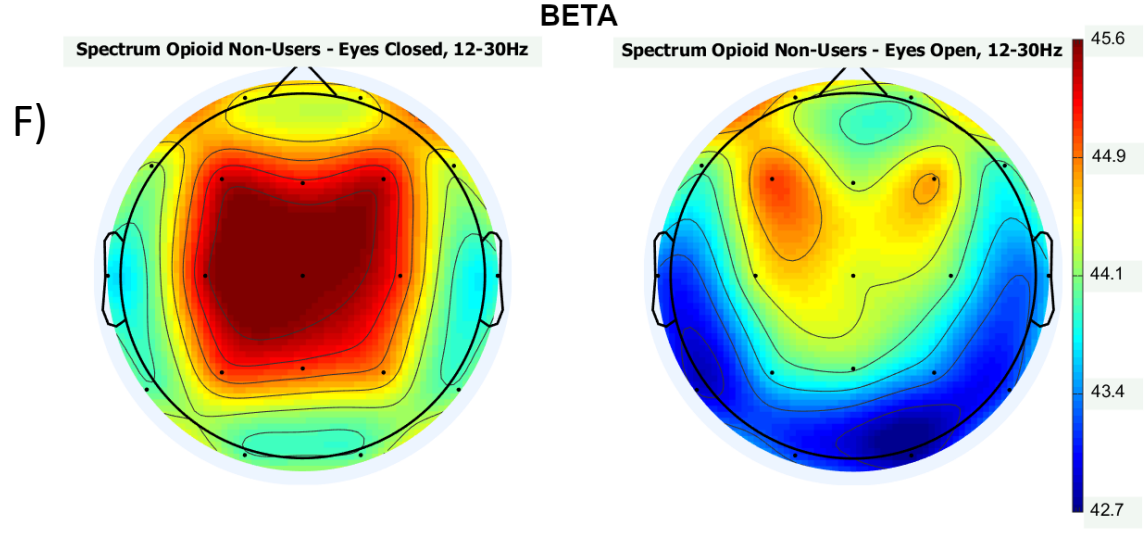

Supplement: Supplementary file 2 — Supplementary Information 2. [file 41598_2021_1982_MOESM2_ESM.pdf]
